# Relationship between rumen microbiota and pregnancy toxemia in ewes

**DOI:** 10.3389/fvets.2024.1472334

**Published:** 2024-09-27

**Authors:** Jiaxin Chen, Siwei Wang, Xuejiao Yin, Chunhui Duan, Jinhui Li, Yue-qin Liu, Yingjie Zhang

**Affiliations:** ^1^College of Animal Science and Technology, Hebei Agricultural University, Baoding, China; ^2^Hebei Key Laboratory of Crop Cultivation Physiology and Green Production, Institute of Cereal and Oil Crops, Hebei Academy of Agriculture and Forestry Sciences, Shijiazhuang, China; ^3^College of Animal Science and Technology, Hebei Normal University of Science and Technology, Qinhuangdao, China

**Keywords:** ewes, pregnancy toxemia, rumen microbiota, rumen fermentation, serum indices

## Abstract

**Introduction:**

Pregnancy toxemia (PT) is a nutritional metabolic disease of ewes in late pregnancy. This study aimed to reveal the relationship between rumen microbiota and PT.

**Methods:**

We selected 10 healthy ewes (CON) and 10 pregnancy toxemia ewes (PT) at 135 days of gestation according to the blood β-hydroxybutyrate (BHBA), glucose (Glu) concentrations and clinical symptoms. Blood and rumen fluid were collected before morning feeding to determine serum biochemical indices and rumen fermentation parameters. Total DNA of rumen fluid was extracted and the V3-V4 regions of 16S rRNA were amplified by PCR for high-throughput sequencing.

**Results:**

The results showed that the serum concentrations of Glu, total cholesterol (TC), low-density lipoprotein cholesterol (LDL-C), uric acid (UA), creatinine (Cr), acetate, propionate, butyrate, and microbial crude protein (MCP) were decreased (*p* < 0.05) and the concentrations of BHBA, aspartate aminotransferase (AST), acetate to propionate ratio (A/P), and ammonia nitrogen (NH_3_-N)were higher (*p* < 0.05) in PT ewes than those in CON ewes. 16S rRNA analysis showed the differences of β-diversity were observed in rumen microbiota between CON and PT ewes. At the phylum level, the relative abundance of Bacteroidota and Proteobacteria were higher (*p* < 0.01), while Firmicutes was lower (*p* < 0.01) in PT ewes. At the genus level, the relative *Prevotella*, *Butyrivibrio*, *Ruminococcus*, *Lachnospiraceae_AC2044_group*, *Lachnospiraceae_XPB1014_group*, *Lachnospiraceae_ND3007_group*, and *Oribacterium* were lower (*p* < 0.01) in PT ewes. Meanwhile, the relative abundance of Oribacterium, Butyrivibrio, Ruminococcus, and Lachnospiraceae_AC2044_group were positively correlated (*p* < 0.01) with Glu, INS, acetate, propionate, and butyrate, and negatively correlated (*p* < 0.01) with BHBA, P, GC, AST, and A/P.

**Discussion:**

In conclusion, the decrease of *Oribacterium*, *Butyrivibrio, Ruminococcus*, and *Lachnospiraceae_AC2044_group* in the rumen of PT ewes reduced the concentrations of volatile fatty acids (acetate, propionate, and butyrate) and serum Glu, and increased BHBA concentration, indicating that the differences in rumen bacteria genera were related to pregnancy toxemia of ewes.

## 1 Introduction

Pregnancy toxemia (PT) is a common and potentially catastrophic nutritional metabolic disorder that occurs with a limited number of individuals or as a flock outbreak, depending on farm feeding management and environmental conditions (1). The clinical diagnosis is based on hypoglycemia and hyperketonemia, which is characterized by loss of appetite, hobbling, and muscle tremors (2). The incidence of PT in sheep is 5 to 20% higher than in goats, and the consequences of pregnancy toxemia are critical, with a loss of 80% of both ewes and offspring (3).

The main cause of PT is nutritional deficiencies in late pregnancy ewes (4). The energy requirements increase by 180 and 240% for ewes carrying twins and triplets, respectively (1). Additionally, the continued growth of the fetus reduces rumen volume, which can lead to insufficient energy intake. As a result, PT is associated with an imbalance in glucose metabolism, resulting in the disruption of the metabolism of carbohydrates, lipids, proteins, and other nutrients, leading to hypoglycemia and disease. Moreover, metabolic homeostasis may be further disrupted by intermittent changes in metabolic state (5, 6). In negative energy balance (NEB), ewes mobilize the body’s energy reserves through an adaptive regulatory mechanism to meet the energy requirements of fetal development; however, excessive body fat mobilization will cause cellular damage to tissues and will accelerate the production of ketone bodies (7). The peroxidation of polyunsaturated fatty acids caused the production of a large amount of reactive oxygen species leading to oxidative stress, which promoted the release of pro-inflammatory cytokines leading to tissue damage (8). Therefore, the detection of serum biochemical indices in ewes is helpful for the early detection and treatment of PT. In addition, long-term NEB in ewes can inhibit cellular DNA replication and cycling to affect fetal development (5, 6). The occurrence of PT is related to the body condition of ewes; obese ewes with a body condition score (BCS) >4 have more adipose tissue to mobilize when dry matter intake (DMI) decreases, which increases the risk of hyperketonemia (9), while lean ewes are more susceptible to starvation pregnancy toxemia due to malnutrition from carrying high multiples (1), and multiparous ewes may have increased difficulty in producing glucose and clearing ketone bodies, which makes ewes more susceptible to PT (10). Metabolic acidosis, hypocalcemia, hypoproteinemia, and dehydration may be secondary to PT (11). When ewes suffer from chronic starvation, the glucose produced by liver gluconeogenesis accounts for approximately 90% of circulating glucose (12), while propionate produced by rumen fermentation is the main substrate source of liver gluconeogenesis in ruminants (13). Rumen fermentation parameters may not only reflect the rumen health status of ewes but also reflect the rumen microbial degradation of ingested feed and the nutrient utilization ability of the body. During strict feeding restriction, rumen epithelium is affected by feed intake to decrease rumen fermentation parameters and change the composition of rumen epithelial microbiota. Meanwhile, during severe feeding restriction, colon bacterial diversity and composition of pregnant ewes are changed, thus changing the colon fermentation pattern (14). The decrease of DMI in dairy cows disrupts the homeostasis of the gastrointestinal microbiota (15), and remodeling the gastrointestinal microbiota by microbiota transplantation can improve feed utilization of low-efficiency cattle (16). These studies also confirmed that the rumen microbiome is associated with nutrient intake, digestion, and absorption.

We found that there are differences in the probability of pregnancy toxemia among ewes carrying the same multiples under the same environment and rearing management, and we speculated that the PT may be related to the differences in nutrient metabolism and microbiota of ewes. Therefore, this study aimed to elucidate the relationship between pregnancy toxemia and rumen microbiota by measuring blood biochemical indices, rumen fermentation parameters, and bacterial community and to provide technical guidance for the prevention and nutritional regulation of pregnancy toxemia in the future.

## 2 Materials and methods

### 2.1 Animals and experimental design

This experiment was conducted between September and November 2020 at Lanhai Farm (Zhangjiakou, China). One hundred healthy pregnant ewes (Hu sheep) of the same parity were observed. Clinical symptoms such as loss of appetite and instability of standing were recorded from the 120th day of gestation. Twenty ewes carrying triplets (litter size was determined by transabdominal ultrasonography, HS-1600 V-7.5 MHz, Japan) were selected as healthy [CON, *n* = 10, β-hydroxybutyrate (BHBA) ≤1.2 mM, glucose (Glu) >3 mM] and pregnancy toxemia (PT, *n* = 10, BHBA ≥3.0 mM, Glu ≤2.5 mM) groups according to the blood BHBA and Glu concentrations and clinical symptoms at 135 days of gestation (1). The experimental ewes were individually kept in pens (2.0 × 1.3 m^2^) provided with free access to water and were uniformly fed and managed. The same total mixed ratio (TMR) was formulated based on NRC (17) recommendations and fed daily at 8:00 am and 5:00 pm with approximately 5% feeding refusal. The DMI recorded for the experimental ewes ranged from 120 days to 135 days of gestation, and the composition and nutrient content of the diets are provided in Table 1.

**Table 1:** Diet composition and nutrient levels of the basal diet (dry matter basis).

Ingredients	Content (%)
Corn stalk silage	38.46
Peanut vine	15.38
Green hay	15.38
Corn	14.38
Bran	3.13
Soybean meal	11.65
CaHPO_4_	0.13
Premix^a^	0.67
Sodium bicarbonate	0.40
NaCl	0.42

Nutritional indicators	Nutritional levels (%)
Metabolic energy, ME, MJ/kg DM^b^	9.40
Crude protein, CP	13.49
Ether extract, EE	2.60
Crude ash	11.43
Neutral detergent fiber, NDF	43.16
Acid detergent fiber, ADF	18.91
Calcium, Ca	0.66
Phosphorus, P	0.33

a Provided per kilogram of feed (dry matter): vitamin A 4402 IU, vitamin D 755 IU, vitamin E 126 IU, Cu 12.50 mg, Mn 28.30 mg, Zn 37.74 mg, Fe 40.88 mg, Co 0.85 mg, I 0.97 mg, and Se 0.85 mg.

b ME is the calculated value, and other nutritional indicators are the measured value.

### 2.2 Sample collection

On day 135 of gestation, serum, and rumen fluid samples were collected from PT ewes and healthy ewes 2 h before morning feeding. Blood samples were collected by the jugular vein sampling method and were centrifuged at 3,000 × g for 10 min. Then, the serum was divided into 1.8 mL frozen storage tubes using a pipette and immediately put into liquid nitrogen tanks, then transported back to the laboratory, and stored in an ultra-low temperature refrigerator. Rumen fluid samples were collected using an oral cannula (Kelibo Animal Husbandry Technology Co., Ltd., Wuhan, China) that reached approximately the bottom of the rumen, taking into account the size of the animal. In the sample collection room, the oral cannula was cleaned with fresh, warm, and distilled water, and the first 20 mL of rumen fluid collected was discarded to avoid saliva contamination. A total of 50 mL of rumen fluid of was stored at −20°C to determine rumen fermentation parameters, and 5 mL were stored at −80°C for DNA extraction.

### 2.3 Serum and rumen fluid parameter measurements

Serum biochemical assay kits (Nanjing Jiancheng Bioengineering Institute, Nanjing, China) were used to determine the concentrations of serum β-hydroxybutyrate (BHBA, β-hydroxybutyrate Assay Kit, E030-1-1), glucose (Glu, Glucose Assay Kit, F006-1-1), non-esterified fatty acids (NEFA, Non-esterified Fatty Acids Assay Kit, A042-2-1), triglycerides (TG, Triglyceride Assay Kit, A110-1-1), total cholesterol (TC, Total cholesterol Assay Kit, A111-1-1), high-density lipoprotein cholesterol (HDL-C, High-density lipoprotein cholesterol Assay Kit, A112-1-1), low-density lipoprotein cholesterol (LDL-C, Low-density lipoprotein cholesterol Assay Kit, A113-1-1), aspartate aminotransferase (AST, Aspartate aminotransferase Assay Kit, C010-2-1), alanine aminotransferase (ALT, Alanine aminotransferase Assay Kit, C009-2-1), alkaline phosphatase (ALP, Alkaline phosphatase Assay Kit, A059-2-2), blood urea nitrogen (BUN, Urea Assay Kit, C013-2-1), total bilirubin (TBIL, Total bilirubin Kit, C019-1-1), uric acid (UA, Uric acid Test Kit, C012-2-1), creatinine (Cr, Creatinine Assay Kit, C011-2-1), calcium (Ca, Calcium Assay Kit, C004-2-1), and phosphorus (P, Phosphate Assay Kit, C006-1-1) according to the manufacturer’s instructions. Insulin (INS, Insulin Assay Kit, H203-1-2), glucagon (GC, Glucagon Assay Kit, H183-1-2), interleukin 1β (IL-1β, Interleukin-1β Assay Kit, H002-1-2), interleukin 2 (IL-2, Interleukin-2 Assay Kit, H003-1-1), interleukin 6 (IL-6, Interleukin-6 Assay Kit, H007-1-2), and tumor necrosis factor-*α* (TNF-α, Tumor Necrosis Factor-α Assay Kit, H052-1-2) were determined using the ELISA test kits (Nanjing Jiancheng Bioengineering Institute, Nanjing, China) according to the manufacturer’s instructions. Serum total protein (TP, Total Protein Quantitative Assay Kit, A045-3-2) and albumin (ALB, Albumin Assay Kit, A028-1-1) concentrations were determined by a colorimetric method using a commercial kit (Nanjing Jiancheng Bioengineering Institute, Nanjing, China).

The rumen fluid samples were immediately analyzed using an electronic pH meter (PHS-3C; Nanjing Nanda Analytical Instrument Application Research Institute). The concentration of the ammonia nitrogen (NH_3_-N) was determined by phenol sodium hypochlorite colorimetry (18) and using a UV spectrophotometer (UV1100; Shanghai Tyco Instruments Co., Shanghai, China) according to the methods of AOAC (2006). The concentration of the microbial crude protein (MCP) was determined by the Coomassie brilliant blue method. The rumen fluid was divided into 2 mL aseptic tubes and centrifuged at 3,000 × g at 4°C for 15 min, and then the supernatant was mixed with 0.2 mL metaphosphoric acid solution (250 g/L) for 30 min. The supernatant was collected and centrifuged at 10,000 × g at 4°C for 10 min for the determination of rumen volatile fatty acids (VFAs). Acetate, propionate, and butyrate concentrations were determined by gas chromatography (Varian 450, Agilent Technologies China, Co., Ltd., China) using 2-ethylbutyric acid as an internal standard. In brief, using H_2_ as the carrier gas with a 30 m × 320 μm × 0.5 μm capillary column (AT-FFAP), the detector temperature was set at 250°C, and the inlet temperature was set at 220°C.

### 2.4 DNA extraction, high-throughput sequencing, and bioinformatic analysis

Rumen fluids were randomly selected from each group for thawing, 1.5 mL of each sample was taken, and a DNA extraction kit (MoBio Laboratories, Carlsbad, CA, United States) was used to extract rumen microbial DNA according to the manufacturer’s instructions. NanoDrop 2000 (Thermo Fisher Scientific, Waltham, MA, United States) and 1% agarose gel electrophoresis were used to check the DNA concentration and quality. PCR universal primers (341F: 5′-GTGCCAGCMGCCGCGGTAA-3′ and 806R: 5′-GGACTACNNGGGTATCTAAT-3′) were designed to amplify the V3–V4 hypervariable region of the 16S rRNA gene based on the microbial genome. The diluted sample DNA was used as a template for PCR amplification, which was carried out on a Mastercycler Gradient (Eppendorf, Hamburg, Germany) in 25 μL reaction volumes containing 12.5 μL of 2 × Taq Plus Master Mix, 1 μL of forward primer (5 μM), 1 μL reverse primer (5 μM), 5.5 μL of ddH_2_O, and 5 μL of DNA (total template quantity was 50 ng). Reaction parameters: initial denaturation at 95°C for 3 min; 35 cycles of denaturation at 94°C for 45 s, annealing at 50°C for 60 s, and elongation at 72°C for 45 s; and stretch for 10 min at 72°C.

The raw data were qualitatively filtered and analyzed using Quantitative Insights into Microbial Ecology (QIIME, version 1.8.0). Sequences with a length greater than 200 bp and an average base mass fraction greater than 25 were labeled as high-quality sequences for subsequent analysis. A total of 620,560 and 527,283 raw tags were obtained for the CON and PT groups, with 607,661 and 519,535 clean tags, respectively. Then, operational taxonomic units (OTUs) were *de novo*-clustered with a 97% sequence similarity cutoff using UPARSE (version 7.1). Finally, 2,056 and 1,689 OTUs were obtained for the CON and PT groups, respectively. The QIIME2 was used to calculate alpha diversity and beta diversity. Alpha diversity included Chao1, observed_species, PD_whole tree, and Shannon indices, which were used to determine the richness and diversity of the bacterial communities. Principal coordinate analysis (PCoA) was used to compare groups of samples based on Bray–Curtis distances and to assess significant differences.

### 2.5 Statistical analysis

Individual ewes were used as experimental units, and the normal distribution of the data was confirmed by the Shapiro–Wilk test. SPSS 22.0 software (SPSS Inc. Chicago, IL, United States) was used to conduct independent samples *t*-test for serum biochemical indices and rumen fermentation parameters of ewes in the CON and PT groups. According to the relative abundance of rumen microbiota, the Wilcoxon rank-sum test was used to test the significance of differences between groups at the phylum and genus levels, respectively. *p* < 0.05 was considered statistically significant in Spearman’s correlation analysis of serum biochemical indices, fermentation parameters, and rumen bacteria (relative abundance >1%).

## 3 Results

### 3.1 Difference in dry matter intake between CON and PT ewes

The DMI of ewes from 120 to 135 days of gestation is shown in Table 2, and there was no difference (*p* > 0.05) in DMI between the PT and the CON ewes.

**Table 2:** Dry matter intake of ewes from 120 to 135 days of gestation.

Item^1^	Group		SEM	*p*-value
	CON (*n* = 10)	PT (*n* = 10)		
DMI, kg/d	1.27	1.20	0.027	0.076

1 DMI, dry matter intake. CON, healthy group; PT, pregnancy toxemia group.

### 3.2 Differences in serum biochemical indices between CON and PT ewes

The serum biochemical indices for CON and PT ewes are shown in Table 3. The concentrations of Glu, TC, LDL-C, INS, UA, Cr, IL-6, and TNF-*α* in the PT group were lower (*p* < 0.05) than those in the CON group, and BHBA, GC, AST, and P were higher (*p* < 0.05) than those in the CON group. The concentrations of NEFA, TG, HDL-C, BUN, TBIL, TP, ALB, ALT, ALP, IL-1β, and IL-2, and there was no difference (*p* > 0.05) in Ca concentration between the two groups.

**Table 3:** Differences in serum biochemical indices between CON and PT ewes.

Item^1^	Group		SEM	*p*-value^2^
	CON (*n* = 10)	PT (*n* = 10)		
BHBA, mmol/L	0.54^b^	5.07^a^	0.118	<0.001
Glu, mmol/L	3.49^a^	1.59^b^	0.142	<0.001
NEFA, mmol/L	0.87	1.03	0.099	0.151
TG, mmol/L	0.40	0.36	0.025	0.145
TC, mmol/L	2.40^a^	1.65^b^	0.125	<0.001
HDL-C, mmol/L	0.83	0.79	0.037	0.263
LDL-C, mmol/L	0.51^a^	0.28^b^	0.062	0.006
INS, mIU/L	28.42^a^	20.44^b^	0.944	<0.001
GC, μg/L	258.64^b^	302.01^a^	7.771	<0.001
BUN, mmol/L	5.66	7.36	0.802	0.079
TBIL, μmol/L	5.63	6.61	0.782	0.242
TP, g/L	65.24	69.75	3.205	0.197
ALB, g/L	32.45	29.55	2.329	0.248
ALT, U/L	3.98	4.03	0.520	0.926
AST, U/L	11.11^b^	22.15^a^	0.798	<0.001
ALP, U/L	14.05	9.66	1.703	0.081
UA, μmol/L	138.32^a^	99.18^b^	3.385	<0.001
Cr, μmol/L	85.08^a^	74.46^b^	5.647	0.042
IL-1β, ng/L	26.97	30.53	1.813	0.085
IL-2, ng/L	38.38	36.61	1.440	0.803
IL-6, ng/L	84.91^a^	74.75^b^	3.091	0.011
TNF-α, ng/L	95.96^a^	79.16^b^	3.734	0.002
Ca, mmol/L	2.15	2.08	0.448	0.131
P, mmol/L	1.63^b^	2.07^a^	0.072	<0.001

1 BHBA, β-hydroxybutyrate; Glu, glucose; NEFA, non-esterified fatty acid; TG, triglycerides; TC, total cholesterol; HDL-C, high-density lipoprotein cholesterol; LDL-C, low-density lipoprotein cholesterol; INS, insulin; GC, glucagon; BUN, blood urea nitrogen; TBIL, total bilirubin; TP, total protein; ALB, albumin; AST, aspartate aminotransferase; ALT, alanine aminotransferase; ALP, alkaline phosphatase; IL, interleukin; TNF-α, tumor necrosis factor-α; UA, uric acid; Cr, creatinine; Ca, calcium; P, phosphorus. CON, healthy group, PT, pregnancy toxemia group.

2 ^a,bValues within a row with different superscripts differ significantly at p < 0.05.^

### 3.3 Difference in ruminal fermentation parameters between CON and PT ewes

The rumen fermentation parameters of CON and PT ewes are shown in Table 4. Compared to the CON group, the concentrations of acetate, propionate, butyrate, and MCP decreased (*p* < 0.01) in the PT group, while A/P and NH_3_-N increased (*p* < 0.05). There was no difference (*p* > 0.05) in rumen pH between the two groups.

**Table 4:** Differences in rumen fermentation parameters between CON and PT ewes.

Item^1^	Group		SEM	*p*-value^2^
	CON (*n* = 10)	PT (*n* = 10)		
Acetate, mmol/L	51.20^a^	27.88^b^	1.589	<0.001
Propionate, mmol/L	17.11^a^	7.73^b^	0.481	<0.001
Butyrate, mmol/L	6.99^a^	3.62^b^	0.375	<0.001
A/P	2.99^b^	3.66^a^	0.216	0.006
Rumen pH	6.94	6.92	0.079	0.803
NH_3_-N, mg/dL	7.67^b^	11.41^a^	0.892	<0.001
MCP, mg/mL	0.55^a^	0.51^b^	0.017	0.023

1 A/P, acetate-to-propionate ratio; NH_3_-N, ammonia nitrogen; MCP, microbial crude protein. CON, healthy group; PT, pregnancy toxemia group.

2 ^a,bValues within a row with different superscripts differ significantly at p < 0.05.^

### 3.4 Difference in rumen microbiota between CON and PT ewes

In this study, 519,535 and 607,661 clean reads were obtained in the CON and PT groups, respectively, with an average of 86,589 and 101,276 clean reads *per ewe* in the CON and PT groups. Rarefaction curves were constructed to quantify the OUT coverage of the sample, as shown in Supplementary Figure S1. All curves approached a plateau, indicating that the sampling depth was sufficient.

**Figure 1:**
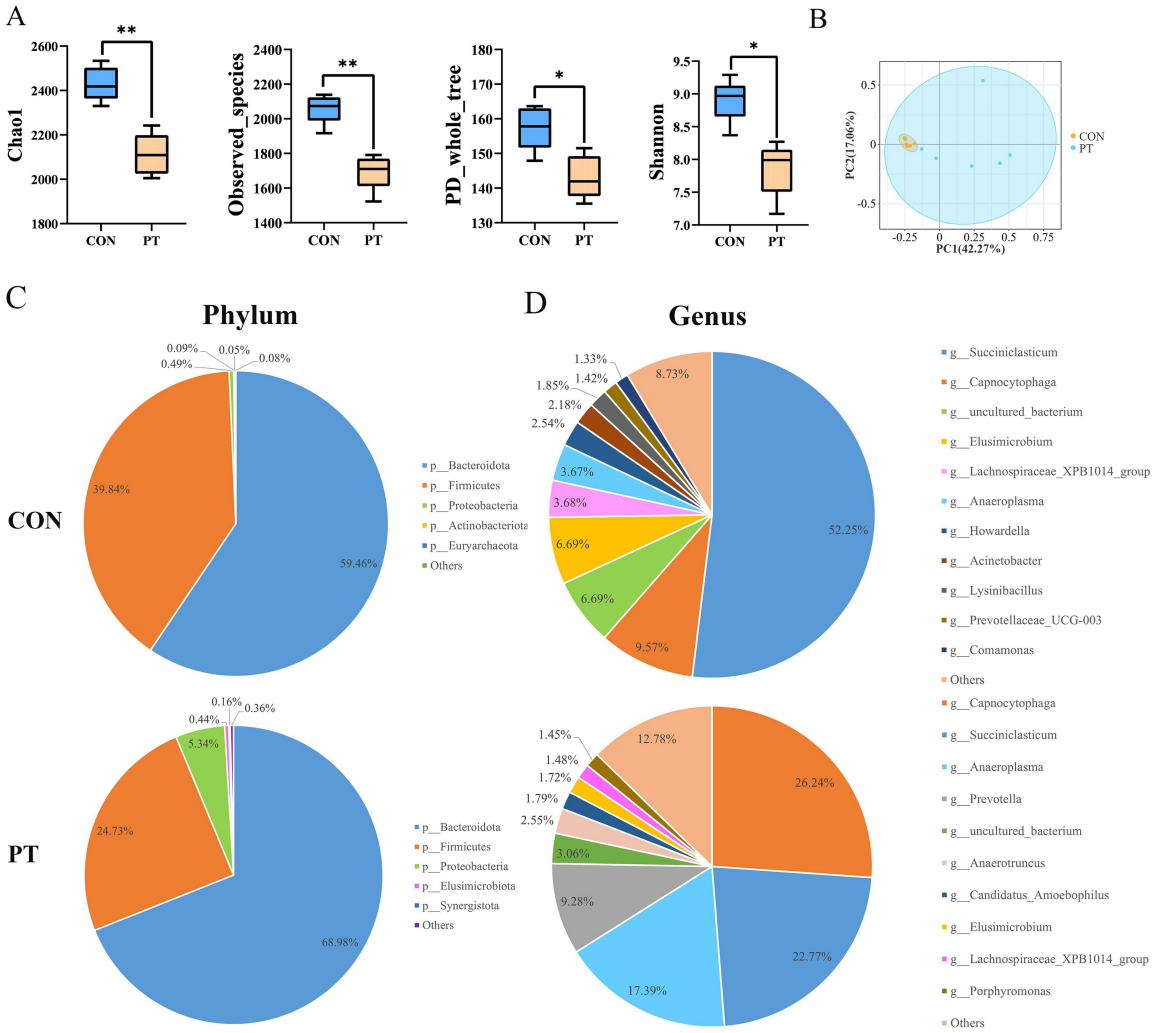
Differences in rumen bacterial communities between CON and PT ewes. **(A)** Alpha diversity including Chao1, observed_species, PD_whole_tree, and Shannon indices. **(B)** Principal coordinate analysis (PCoA) of differences in bacterial community composition, the percentage represents the relative contribution of two principal coordinates (PC1–PC2). **(C,D)** Rumen bacterial community composition in groups CON and PT at phylum **(C)** and genus levels **(D)**. CON, healthy group (*n* = 6), PT, pregnancy toxemia group (*n* = 6).

The alpha diversity analysis showed significant differences in the Chao1, observed_species, Shannon, and PD_whole tree indices between the two groups (*p* < 0.05). The rumen microbiota richness and diversity varied significantly within the groups (Figure 1A). The PCoA results showed that PT ewes reduced the intra-group similarity based on Bray–Curtis distance (ANOSIM: *R* = 0.494, *p*-value = 0.004), indicating poor stability of rumen bacteria in the PT group (Figure 1B).

To further study the classification and composition of rumen bacteria, we compared the relative abundance of rumen microbiota in the two groups at the phylum and genus level (Figures 1C,D). Bacteroidota and Firmicutes were the most abundant in the rumen of the two groups of ewes at the phylum level. The proportions of Bacteroidota (68.98%) and Proteobacteria (5.34%) in the PT group were increased, while the proportion of Firmicutes (24.73%) was decreased (Figure 1C). The dominant bacterial genera in the CON group were *Succiniclasticum* (52.25%), *Capnocytophaga* (9.57%), *uncultured_bacterium* (6.69%), and *Elusimicrobium* (6.69%). The species and proportions of dominant bacteria in the PT group were changed and were dominated by *Capnocytophaga* (26.24%), *Succiniclasticum* (22.77%), *Anaeroplasma* (17.39%), and *Prevotella* (9.28%) (Figure 1D).

**Figure 2:**
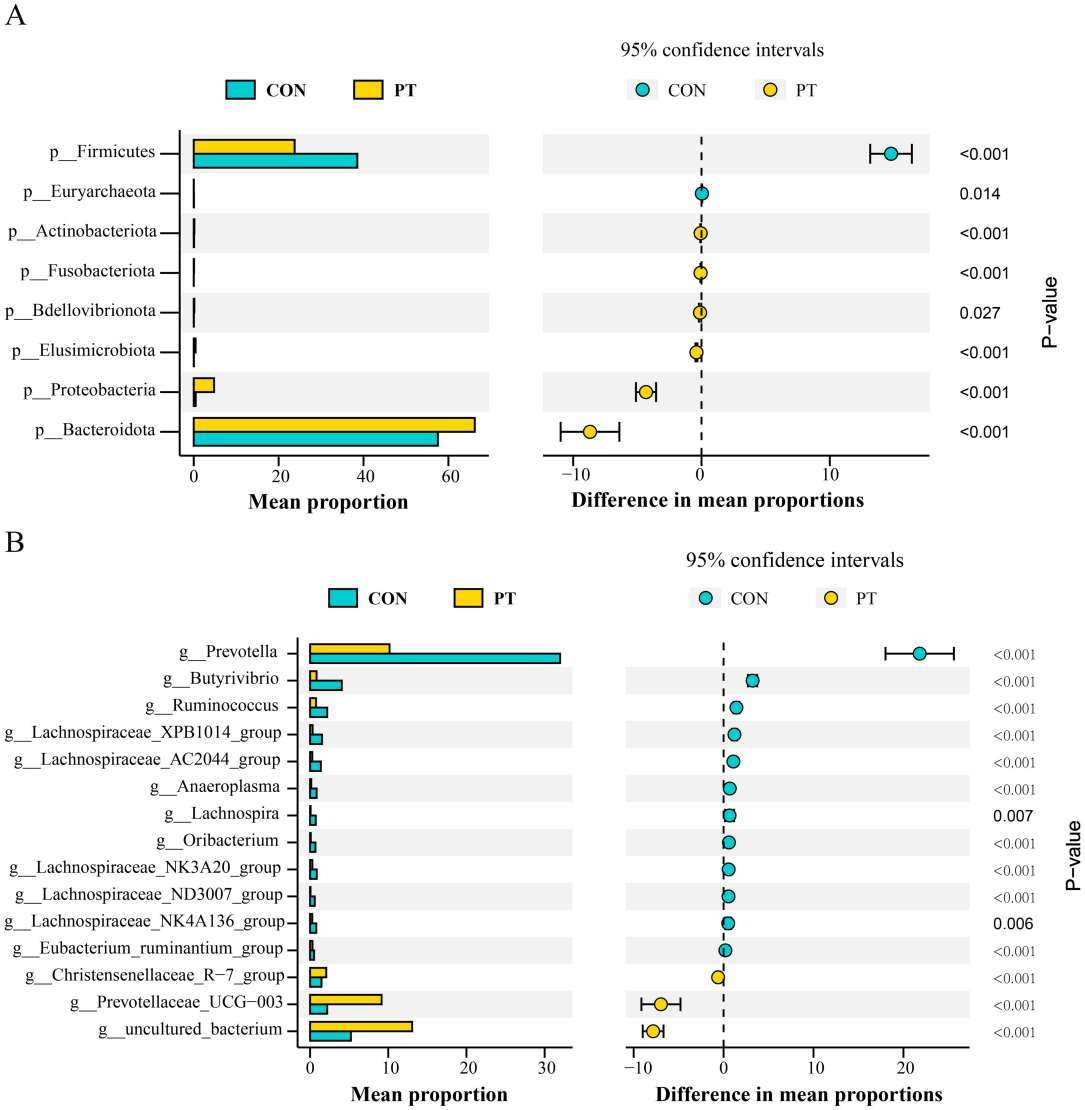
Differences in bacterial classification and proportion in CON and PT groups at the phylum **(A)** and genus levels **(B)**. CON, healthy group (*n* = 6), PT, pregnancy toxemia group (*n* = 6).

A total of 23 phyla, 41 classes, 82 orders, 145 families, and 258 genera were identified in this study. We selected phyla and genera with a relative abundance greater than 1% for subsequent analysis. At the phylum level, the relative abundance of Bacteroidota, Proteobacteria, Fusobacteriota, Bdellovibrionota, Elusimicrobiota, and Actinobacteriota in the PT group was higher (*p* < 0.01) than those in the CON group. On the contrary, the relative abundance of Euryarchaeota and Firmicutes decreased (*p* < 0.01) in the PT group (Figure 2A). At the genus level, the relative abundance of *uncultured_bacterium*, *Prevotellaceae_UCG-003*, and *Christensenellaceae_R-7_group* in the rumen of ewes in the PT group increased (*p* < 0.01); however, the relative abundance of *Prevotella*, *Butyrivibrio*, *Ruminococcus*, *Lachnospiraceae_XPB1014_group*, *LachnospiraceaeAC2044group*, *Anaeroplasma*, *Lachnospira*, *Oribacterium*, *Lachnospiraceae_NK3A20_group*, *Lachnospiraceae_ND3007_group*, *Lachnospiraceae_NK4A136_group*, and *Eubacterium_ruminantium_group* in the PT group decreased (*p* < 0.01) than those in the CON group (Figure 2B).

### 3.5 Correlation analysis between ruminal microbiota, serum biochemical indices, and rumen fermentation parameters

**Figure 3:**
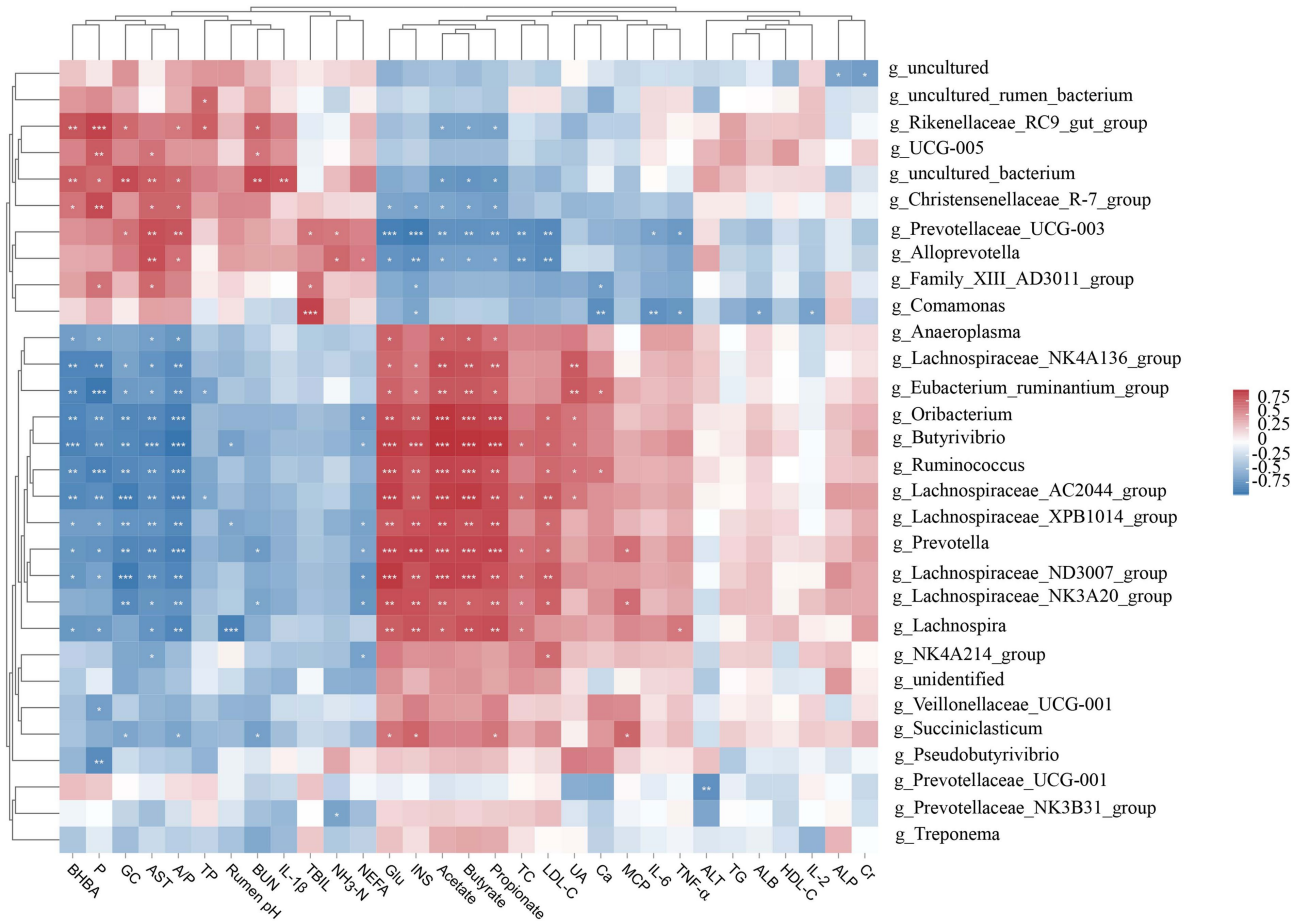
Correlation analysis between the ruminal microbiota (relative abundance >1%), serum biochemical indices, and rumen fermentation parameters. Glu, glucose; BHBA, β-hydroxybutyrate; NEFA, non-esterified fatty acids; TG, triglycerides; TC, total cholesterol; HDL-C, high-density lipoprotein cholesterol; LDL-C, low-density lipoprotein cholesterol; AST, aspartate aminotransferase; ALT, alanine aminotransferase; ALP, alkaline phosphatase; BUN, blood urea nitrogen; TBIL, total bilirubin; Ca, calcium; P, phosphorus; INS, insulin; GC, glucagon; UA, uric acid; Cr, creatinine; IL, interleukin; TNF-*α*, tumor necrosis factor-α; TP, total protein; ALB, albumin; NH_3_-N, ammonia nitrogen; MCP, microbial crude protein; A/P, acetate-to-propionate ratio. ^*^p < 0.05, ^**^p < 0.01, and ^***^p < 0.001.

To explore how rumen microbiota affects host metabolism, we established a Pearson correlation matrix to explore the relationship between them. We found that most of the rumen bacterial genera were correlated with serum biochemical indicators and rumen fermentation parameters (Figure 3). The relative abundances of *Oribacterium*, *Butyrivibrio*, *Ruminococcus*, and *Lachnospiraceae_AC2044_group* were positively correlated (*p* < 0.01) with Glu, INS, acetate, propionate, and butyrate and negatively correlated (*p* < 0.01) with BHBA, P, GC, AST, and A/P. *Lachnospiraceae_NK4A136_group*, *Eubacterium_ruminantium_group*, *Lachnospiraceae_XPB1014_group*, *Prevotella*, and *Lachnospiraceae_ND3007_group* were positively correlated (*p* < 0.05) with Glu, INS, acetate, propionate, and butyrate and negatively correlated (*p* < 0.05) with BHBA, P, GC, AST, and A/P. In contrast, the relative abundances of *Christensenellaceae_R-7_group*, *Prevotellaceae_UCG-003*, and *Alloprevotella* were negatively correlated (*p* < 0.05) with Glu, INS, acetate, propionate, and butyrate and positively correlated (*p* < 0.05) with AST and A/P. The relative abundances of *Rikenellaceae_RC9_gut_group* and *uncultured_bacterium* were negatively correlated (*p* < 0.05) with acetate, propionate, and butyrate and positively correlated (*p* < 0.05) with BHBA, P, GC, A/P, and BUN.

## 4 Discussion

Pregnancy toxemia is defined as a metabolic disorder of energy deficiency, with clinical symptoms most often presenting in the last 3 weeks of gestation, although some may occur earlier depending on the nutritional status of the ewes (1). The basic disorder of pregnancy toxemia is an imbalance in maternal glucose homeostasis, which leads to NEB characterized by hypoglycemia and hyperketonemia (19). However, the pathogenesis of glucose homeostasis is still unclear. The exponential growth of the fetus in late pregnancy, approximately 70% of which occurs in the last 30–40 days, exacerbates glucose loss (20, 21). Fetal glucose utilization is completely dependent on maternal glucose availability given the inability of the fetal liver to synthesize glucose (22, 23); in contrast to the facilitated diffusion of glucose, amino acids are actively transported into the placenta (24), so that in response to maternal hypoglycemia, there is a shift to amino acid oxidation to compensate for the energy substrate deficit and alleviate fetal hypoglycemia (25). Our results found that the DMI of PT ewes between 120 and 135 days of gestation was similar to that of healthy ewes. Both groups had DMI levels below the recommended requirements (17), leading us to infer that the experimental ewes experienced NEB during late gestation. When confronted with nutritional limitations, pregnant animals mobilize body fat and labile body proteins to support gluconeogenesis and supply amino acids (26). The metabolites produced from lipid mobilization enter the body tissues as energy substrates in three ways: most enter the tricarboxylic acid cycle for complete oxidation and energy supply, while some are esterified to triglycerides (TG). An accumulation of TG in the liver increases the risk of steatosis. Additionally, a portion of these metabolites is oxidized to ketone bodies (27, 28). Lipidolysis in late pregnancy promotes elevated NEFA, which is associated with insulin response and peripheral insulin resistance, and ketogenesis is the result of reduced glucose and insulin concentrations (29). In our study, we observed a decrease in serum glucose and insulin and an increase in BHBA in ewes with PT. High concentrations of BHBA inhibit gluconeogenic pathways and exacerbate NEB (30). Elevated BHBA levels also reduce the mobilization of adipose tissue and the release of NEFA, which in turn reduces the production of ketone bodies (31). Our study found no difference in NEFA and TG between the two groups. This lack of difference may be attributed to the high concentrations of BHBA in ewes with pregnant toxemia, which may inhibit the production of NEFA and TG. An interesting observation from a related study is that after a 36 h fast in healthy humans, liver lipid content increases, although the degree of lipid accumulation can vary (32). C57BL/6J and SJL/J mice differ dramatically in their susceptibility to fast-induced hepatic steatosis. Although both strains of mice lost 60% of their body TG after a 24-h fast, SJL/J mice did not develop steatosis. In these mice, a greater proportion of the NEFA produced from lipid mobilization was absorbed by the heart and skeletal muscle (33). We found that healthy and PT ewes exhibited differences in glycolipid metabolism, which may lead to variations in ketone body production, indicating that lipid oxidation and utilization processes differ between individuals.

The recommended requirements for energy, crude protein, calcium, and phosphorus for ewes in late gestation was 1.5 to 2 times than that of ewes in early gestation (17), and with the increase of gestation days, ewes’ demand for energy and protein increased (34). At first, it was thought that feed intake in ruminants was regulated by glucose receptors in the liver, known as the hepatic oxidation theory (HOT) (35). In recent years, researchers have found that rumen fermentation has been used to understand the complexity of feed intake metabolism regulation in ruminants (36, 37). The ratio of non-fiber carbohydrates (NFC) to neutral detergent fiber (NDF) alters the composition of the rumen microbiota, and its imbalance also induces rumen acidosis in ewes (38), whereas the NDF content of feeds is an important regulator of feed intake in dairy cows (39). However, the ability of pregnant cows to consume NDF is reduced with the number of gestation days and gestation times (1). Previous studies have found that the degradation of NDF in ewes is mainly affected by the microbiota (40); moreover, Wang et al. (41) found that rumen microbiota affected short-chain fatty acid metabolism and BHBA accumulation may be related to ketosis even when feed intake was sufficient. Thus, we speculated that the metabolic differences of ewes in late gestation may be related to the rumen microbiota involved in nutrient degradation. NH_3_-N is the end product produced by the decomposition of nitrogen-containing substances in the rumen bacterial community, and MCP is the conversion of NH_3_-N that can provide 50–80% absorbable protein for ruminants (42). Interestingly, we found that the ability of PT ewes to produce NH_3_-N was increased; however, the ability of the rumen microbiota to synthesize MCP using ammonia was decreased. One study found that 5 mg/dL is considered the optimal concentration of NH_3_-N synthetic microbial protein, and when NH_3_-N content is reduced, the serum BUN is transferred to the rumen to alleviate amino-nitrogen deficiency, and defaunation also reduces the NH_3_-N content (43). Our study found that BUN was not different between the two groups, while the content of NH_3_-N in PT ewes was increased, which may be related to the relative abundance of rumen protozoa. Richness and diversity can be used as indicators of bacterial community function in the rumen, and specific microbiomes are associated with metabolic pathways to provide more energy to the host (44). A previous study found that the ketosis states were accompanied by substantial changes in the bacterial taxa in the rumen (41). In this study, we observed a decrease in both the richness and diversity of the rumen microbiota in PT ewes, which may be one reason for their condition. Additionally, there is growing evidence of a link between gastrointestinal microbes and host disease (45, 46). Moreover, fecal microbiota has been shown to be highly informative in predicting human body mass index, glycemic status, and fasting glucose levels (46). For example, metabolic disease (rumen acidosis) is associated with rumen microbial disorders (47). In cows with ketosis, the composition of the rumen microbiome is altered. Propylene glycol treatment has been shown to increase the relative abundance of *Prevotella*, *Succinivibrionaceae_UCG-001*, and *Prevotellaceae_UCG-001*, and to increase propionate production. This treatment can help reduce blood BHBA levels, thereby promoting a healthier state of the rumen microbiota (48). Cows with circulating BHBA concentrations greater than 1.4 mmol/L had a 2.83–6.70 times higher risk of developing secondary left displaced abomasum (49). Additionally, changes in the gut microbiota are associated with ketogenesis and glucose metabolism in cows with left displaced abomasum (50).

The VFA produced by rumen microbial fermentation feed is the main energy source of ruminants (51). Rumen *Oribacterium* and *Lachnospiraceae_AC2044_group* belong to Firmicutes (52), and *Ruminococcus* and *Lachnospiraceae* not only have the ability to degrade cellulose but also contain many plant polysaccharide hydrolase genes (53, 54). *Prevotella* can encode a highly conserved polysaccharide utilization system to degrade soluble gluconic acid and starch polysaccharides and can also use plant cell wall polysaccharides to promote the degradation of xylan (55). In addition, the propionate production capacity of *Prevotella* is 2–3 times higher than that of *Bacteroides* (56). Meanwhile, methane and propionate synthesis can function as the main hydrogen sinks in the rumen, and a strong negative correlation can be detected between these two processes, which provides evidence that Prevotella is associated with low methane and high propionic yield (57). *Butyrivibrio* can degrade hemicellulose and produce butyrate using polysaccharides such as starch, pectin, and xylan as substrates (58). Furthermore, a previous study estimated the association between the abundances of rumen bacterial and archaeal OTUs and milk BHBA and acetone concentrations, and they found that the most strongly correlated bacterial OTUs are dominated by members of the *Prevotellaceae* and *Ruminococcaceae* families, both of which showed a negative correlation with plasma levels of BHBA (59). Our study had similar results, we observed a decrease in the relative abundance of *Prevotella* (Bacteroides), *Butyrivibrio* (Firmicutes), *Ruminococcus* (Firmicutes), and *Lachnospiraceae* (Firmicutes) in PT ewes, and they were positively correlated with the concentrations of Glu, acetate, propionate, and butyrate and negatively correlated with BHBA. Our results indicated that the utilization capacity of feed polysaccharides and hemicelluloses in PT ewes was reduced, and the pathway of VFA production was inhibited, which resulted in an inadequate supply of energy to the ewes in addition to increased fat mobilization and BHBA production. Furthermore, BHBA stimulation of mammary epithelial cells increases the concentration of malondialdehyde, promotes the accumulation of reactive oxygen species, and enhances the expression of inflammatory indicators. This results in oxidative stress and inflammatory response (60). Additionally, *Prevotella* and *Lachnospiraceae* regulate host immunity (61, 62) and can promote colonization resistance to rumen pathogens (63, 64). The negative correlation between these microbial groups and BHBA suggests that the decreased relative abundance of *Prevotella* and *Lachnospiraceae* may be related to the BHBA-induced inflammatory response. Proteobacteria is the dominant phylum endemic to PT ewes and includes well-known opportunistic pathogens such as *E. coli* and *Salmonella* (65). Therefore, the decrease in *Prevotella* and *Lachnospiraceae* and the increase in Proteobacteria promote the accumulation of pathogenic bacteria in the rumen. These changes in the rumen bacteria may be related to the development of PT.

## 5 Conclusion

In this study, we found that the concentrations of serum glucose, acetate, propionate, butyrate, and MCP were lower, while the concentrations of BHBA, AST, A/P, and NH_3_-N were higher in ewes with pregnant toxemia than in healthy ewes. The decreases in the relative abundances of rumen bacteria such as *Oribacterium*, *Prevotella*, *Butyrivibrio*, *Ruminococcus*, and *Lachnospiraceae_AC2044_group* were associated with reduced production of acetate, propionate, and butyrate as well as decreased serum glucose concentrations and increased BHBA and AST concentrations. These findings indicate that differences in rumen bacterial genera are related to pregnancy toxemia in ewes.

## Data Availability

The datasets presented in this study can be found in online repositories. The names of the repository/repositories and accession number(s) can be found in the article/Supplementary material.
