# Supplementary material for: Relationship between rumen microbiota and pregnancy toxemia in ewes
Source: Front Vet Sci. 2024 Sep 27;11:1472334. doi: 10.3389/fvets.2024.1472334 (PMC11466943; doi:10.3389/fvets.2024.1472334)
Supplement: Supplementary file 1 [file Image_1.pdf]

Rumen microbiota related to pregnancy toxemia of ewes

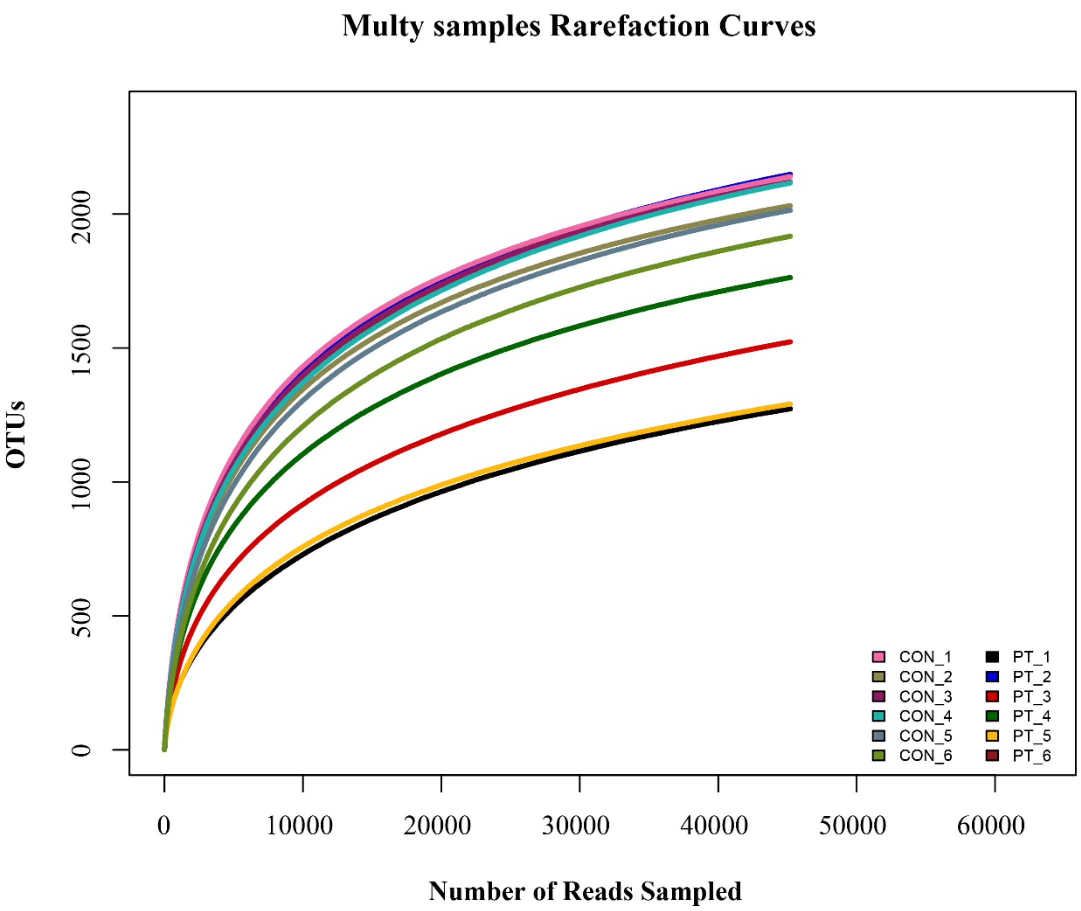

Supplementary Figure S1. Rarefaction curves based on operational taxonomic units (OTUs) for each sample in CON and PT ewes. CON, healthy group (n=6), PT, pregnancy toxemia group (n=6).
